# Diversity matters: Effects of density compensation in pollination service during rainfall shift

**DOI:** 10.1002/ece3.5500

**Published:** 2019-08-18

**Authors:** Ronita Mukherjee, Rittik Deb, Soubadra M. Devy

**Affiliations:** ^1^ Ashoka Trust for Research in Ecology and the Environment Bangalore India; ^2^ Manipal Academy of Higher Education Manipal India; ^3^ National Centre for Biological Sciences Bangalore India

**Keywords:** agricultural yield, bee diversity, climatic variation, drought, fruit‐to‐flower ratio, sustainability, wild pollinators

## Abstract

1. Extreme weather events are increasing in frequency due to the warming climate. Such extremities can jeopardize ecosystem services and create economic imbalances. Tropical developing countries are predicted to suffer the maximum consequences of such events.
2. We examined the impact of such an event—extreme rainfall fluctuation—on a critical ecosystem service—pollination, which can be intricately linked to a country's economy. We performed this study in a dominant peri‐urban vegetable hub of an agriculture‐dependent developing country.
3. We found that the yield of all pollinator‐dependent crops grown across a large spatial scale (district) over multiple years (six) drastically declined with the decrease in rainfall.
4. At the local scale, we found that the dominant crop (representative horticultural crop) had a significant drop in yield during drought, likely due to the production of fewer female flowers and a significant shift in the pollinator community.
5. We found that *Trigona* sp. (one of the four pollinators) was the critical pollinator positively influencing fruit‐to‐flower ratio (FFR) (an indicator of pollination service) in the normal rainfall year. However, despite its sharp decline during drought, the FFR remained unaffected. We found that during drought, *Apis dorsata* was crucial in maintaining FFR and compensated for the decline of the critical pollinator across 67% farmlands.
6. Our study demonstrates the role of ecosystem stabilizing mechanism rescuing the crucial ecosystem service during climatic variability over the temporal scale.

## 1 INTRODUCTION

Global climatic unpredictability is responsible for significant yield variability across cultivated crops (Abewoy, 2018; Giannini et al., 2017; Ray, Gerber, MacDonald, & West, 2015). This yield variability can be attributed to the climatic fluctuations, causing stress (such as water and temperature) on plants (Berman & DeJong, 1996; Hatfield & Prueger, 2015; Nuruddin, Madramootoo, & Dodds, 2003). For pollinator‐dependent crops, yield can also be jeopardized due to climatic variability impacting pollinator communities (Arnold et al., 2018; Glenny, Runyon, & Burkle, 2018; González‐Varo et al., 2013). Increasing climatic extremities (Cai et al., 2014; Thornton, Ericksen, Herrero, & Challinor, 2014; Vasseur et al., 2014) has been shown to impact the complex plant–pollinator relationship (Fontúrbel, Lara, Lobos, & Little, 2018; Potts et al., 2010) that can potentially lead to severe yield instabilities (Klein et al., 2007). During rapid climate change, the increase in such variability will lead to significant ecological and economic impact (Vanbergen & Insect Pollinators Initiative, 2013). The economies of developing countries, mostly contingent on the agricultural yield of their horticultural crops (http://www.fao.org/, www.ishs.org/defining-horticulture), are predicted to bear the maximum consequences (Gallai, Salles, Settele, & Vaissière, 2009).

Global climate change studies reviewing global climate as well as crop growth models have explicitly pointed at the role of precipitation in impacting crop yield (Kang, Khan, & Ma, 2009). Time series crop data across the globe analyzed by Ray et al. (2015) also reveal precipitation inconsistency to impact and drive crop yield significantly. In an agriculturally dependent developing country like India, approximately 60% of the total cropping area is rain‐fed (Mall, Singh, Gupta, Srinivasan, & Rathore, 2006), making agriculture heavily rainfall‐dependent. Highly erratic rainfall pattern since the last century has been predicted to majorly affect the crop growth in India (Mall et al., 2006). Several other such developing nations whose crop production critically depends on rainfall house the majority of the world population (Mendelsohn & Dinar, 1999), making climate‐induced cropping variability a global issue. Hence, it becomes necessary to understand the climate‐sensitive factors that drive the yield decline.

Studies examining the impact of climate change on agricultural yield have predominantly focused on plant traits or functions. Rainfall fluctuation is known to induce instability in agricultural yield (Mendelsohn, 2008). Rainfall deficiency has been shown to increase abscisic acid, reduce starch and carbohydrate in the reproductive stage of plants. These affect crop pollination by disrupting ovary development, increasing pollen grain sterility, and decreasing nectar volume, flower attractiveness, and seed set percentage (Alqudah, Samarah, & Mullen, 2011). Rainfall decline has also been shown to shift the phenology of forests (Peñuelas et al., 2004) and floral abundances (Phillips et al., 2018; Thomson, 2016).

Climatic variation can also influence pollinator communities directly, or indirectly by influencing the plant–pollinator interaction. Severe rainfall decline can cause local pollinator extinction, as shown in diecious fig species of Borneo (Harrison, 2000, 2001). Pollinator distribution and abundance are found to be to largely dependent on rainfall over large geographic scales, with bees dominating xeric climates while flies the wetter ones (Devoto, Medan, & Montaldo, 2005). Any sudden shift in climatic zones can thus create a drastic shift in pollinator community and plant–pollinator interaction across the globe (Hegland, Nielsen, Lázaro, Bjerknes, & Totland, 2009). Additionally, the rainfall decline can also indirectly impact the pollinator populations by affecting the availability of floral resources (Phillips et al., 2018). However, different pollinator species can respond to resource fluctuations differently, some being more sensitive than others. One of the key drivers for such differential response toward resource fluctuation can be attributed to their foraging ranges (Inoue, Nakamura, Salmah, & Abbas, 1993; Itioka et al., 2001; Koeniger & Koeniger, 1980; Nagamitsu & Inoue, 2002).

Though it is important to separately understand the impacts of rainfall shift on plant and its pollinators in agroecosystems, it is essential to examine plants, pollinators, and their interactions together, to understand the actual impact of climatic instabilities on the yield. In this context, we examined how climatic variability (in the form of rainfall variation) covaries with the yield of horticultural crops in an Indian peri‐urban agricultural setting. We examined the likely impact of rainfall decline on the average yield of horticultural crops over a decade across a large spatial scale (district). At a local scale, we examined the likely impact of drastic rainfall decline on the dominant crop's yield. We also examined the potential effect of rainfall decline on plant components, pollinator communities, and their interactions. We hypothesized that significant rainfall decline would have a critical impact on crop yield by influencing both plant and pollinator communities. However, we speculated that the presence of high pollinator diversity in tropics might mitigate the impact of climate change and thus help in sustaining the pollination service and crop yield. We expected that, as different pollinator species can exhibit varying climatic resilience, the presence of diverse pollinator species can act as an ecosystem stabilizing mechanism sustaining pollination service during climatic fluctuation.

## 2 MATERIALS AND METHODS

### 2.1 Study area

**Figure 1:**
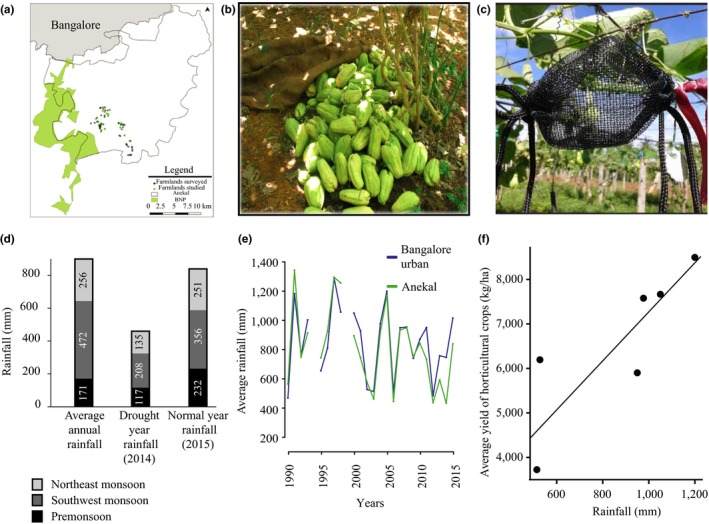
(a) Map of study area showing all the farmlands surveyed (in black) and the farmlands chosen for local‐scale pollination study (in green). The area in gray indicates Bangalore city. The area in green demarcates Bannerghatta National Park (BNP). The area in white demarcates Anekal. (b) Harvested Chayote squash. (c) A bagged flower during our pollinator exclusion study. (d) Bar plot showing the rainfall difference between the normal (2015) and drought (2014) years. (e) Line plot depicting the coherence in rainfall pattern between Bangalore urban district and local scale (Anekal) highlighting severe rainfall fluctuations over the years. (f) Regression plot is showing the dependence of crop yield (*y*‐axis) on rainfall (*x*‐axis). The crops whose yields were used to calculate the average yield were beans, tomato, brinjal, squash, mango, papaya, guava, and sapota. The crop composition remained constant across the years

We chose Bangalore urban district—a fast developing area comprising of several important vegetable hubs catering to southern India—to understand the relationship between rainfall and crop yield. We chose an important vegetable hub—Anekal Taluk (an administrative division of subdistrict)—to perform the fine‐scale observation and experimental studies to delineate the factors that are critical for yield fluctuations during rainfall variation. Anekal—geographically situated at the confluence of a national park and a developing city (Figure 1a and Figure S1)—is known to grow horticultural crops in 73% of its cultivable area (data from Directorate of Economics and Statistics, Bangalore, DES henceforth). We surveyed Anekal with the help of a local guide to examine all the farmlands that grew horticultural crops (*N* = 73; Figure 1a). For each farmland, we noted the existing crop and also interacted with farmers to assess the major crops grown in the farmland. Our survey allowed us to decide on the dominant horticultural crop in Anekal.

### 2.2 Data

#### 2.2.1 Did Bangalore urban district experience rainfall variation at the regional and local scale? Does yield fluctuations covary with rainfall variation?

We collected data on 24 years (spanning over 26 years from 1990 to 2015; out of which 1994 and 1999 are missing) of rainfall at both the district (regional) and subdistrict (local) level from DES. For yield information, we collected 6 years (2000, 2002–2005, and 2007) of pollinator‐dependent crop yield data at district (regional) level. Additionally, for comparison, we also collected 9 years (2000, 2002–2005, 2007, 2010, and 2012–2013) of crop yield data for non‐pollinator‐dependent crop at district (regional) level. These data were collected to examine the rainfall fluctuation and its relationship with crop yield. From our local‐scale survey, we selected Chayote squash (*Sechium edule*) as our study species, the most recurrently grown horticultural crop of the study region (Figure 1b). We randomly selected 24 farmlands spread across Anekal (Figure 1a) for our local‐scale plant–pollinator interaction study. We aimed to examine how various plant characteristics or traits (such as flower number, fruit‐to‐flower ratio, and fruit weight and size), pollinator visitation, and species composition of pollinators covary with rainfall change. For this purpose, we collected paired data for two consecutive years, 2014 and 2015, among which the rainfall amount differed significantly. The rainfall deficit year (2014) received only 460 mm of rainfall—one of the lowest across 24 years—and was declared a severe drought year (by DES; Figure 1d). The normal rainfall year (2015) received 839 mm rainfall—close to average rainfall of this region (Figure 1d).

#### 2.2.2 Which plant traits and pollinators covary with rainfall fluctuations?

Before asking this question, it was critical to understand (a) whether our crop—Chayote squash—is a pollinator‐dependent crop and (b) what is the peak pollinator visitation hour in this crop?

##### Is our target crop pollinator‐dependent?

An earlier study (Heard, 1999) had reported that Chayote squash is a pollinator‐dependent species. We decided to examine the same in our study locality (in a single farmland Field 1, 23–25 October 2014). We examined the pollinator dependence of Chayote squash using pollination exclusion experiment. We successfully tagged 80 buds and observed them until they withered or bore fruit. This treatment acted as our positive control. We also bagged 79 female buds (Kearns & Inouye, 1993) to exclude pollinator access (Figure 1c). We designed two‐side open mesh bags with drawstrings (11.5 cm × 10 cm; Figure 1c) to bag solitary female buds present both at the tip and the middle of the tendril. If Chayote is pollinator‐dependent, we expected to find fruits in the open treatment, whereas no fruit in the bagged treatment.

##### What is the peak pollinator visitation hour in the study species?

We standardized the timing of peak pollinator visitation by conducting observations for three consecutive days in each of the three randomly selected farmlands, from 6 a.m. to 5.30 p.m. continuously. The observation was performed in June 2014 during the peak flowering season of Chayote (June–November). There were four squares selected in each farmland/day. Hence, 12 squares were observed/farmland across 3 days. Two such squares were allotted to one observer each day. Each observer spent 25 min/hr observing visitors in the first square and then shifted to the second one after a 5‐min break. A similar observation was performed in two more farmlands to standardize the peak pollinator visitation time period. We scored any visitor that visited and touched the male or female reproductive parts of a flower. We calculated the average number of visitors/day in each quadrat/farmland. We binned the number of visitors/farmland in hours and plotted these data to determine the peak visitation hours.

##### Examining the plant traits: floral number, fruit‐to‐flower ratio, and fruit weight, length, and width

We measured the male, female, and total flower numbers (all flowers), fruit‐to‐flower ratio (FFR), and fruit weight, length, and width across all the farmlands (*N* = 24) over the two years to examine whether these factors showed significant variation between the study years.

We selected sixteen random quadrats (1 m × 1 m) in each farmland and counted the total number of open male and female flowers in them (see details on site selection in the visitation observation section below). Additionally, we tagged randomly selected 50 open female flowers (using ribbons) in each farmland and revisited them at the next harvest cycle (15th day) to measure the flower to fruit conversion. This fruit‐to‐flower ratio acted as a measure of the efficiency of the plant–pollinator interaction (Ne'Eman, Jürgens, Newstrom‐Lloyd, Potts, & Dafni, 2010). The average fruit quality in each farmland was determined by measuring the length, width, and weight of 10 randomly selected mature fruits. The weight of each fruit was measured in a compact electronic scale (TS 200) with an error (or precision) of 0.5 g. We also measured fruit length and width for the same 10 fruits using an Insize Vernier caliper (model 112–150; Insize USA, Georgia USA). We calculated the average of these 10 fruit weights, lengths, and widths to determine the quality of an average fruit of a farmland.

##### Calculating the farmland yield

We formulated the following equation to calculate the average yield of each farmland/harvest cycle (15 days):

$$\begin{array}{c} \text{Yield measured as the kilogram of fruit produced per hectare}\\ \;\;=\text{The average number of female flowers per quadrat}\left(\text{sq}\text{. m}\right)\\ \;\;\times \;10,000\left(\text{square meter to hectare conversion}\right)\times \text{FFR}\\ \;\;\times \;\text{average fruit weight of the farmland}\left(\text{in grams}\right)/1,000\left(\text{gram to kg conversion}\right). \end{array}$$

We also calculated the confidence interval of the yield for each farm using the variance in the number of female flowers/quadrat and fruit weight of each farmland.

##### Examining the pollinators—visitor type, abundance, and composition

We used two observers in each farmland for two consecutive days to estimate visitation pattern across all the farmlands in the landscape. We recorded the observation dates (Table S1) for all the farmlands across the years.

We designed our visitation observation protocol after surveying all the study farmlands. We divided each farmland into four quarters using the two diagonals, and randomly assigned a diagonal to an observer. Before visitation observation site selection, we left 4 m from all the four diagonal ends. This was performed to control for edge effect on visitation. Each observer on each day selected four quadrats of 1 m × 1 m dimension at random points along the diagonal (Figure S2). A minimum of 20 flowers (male and female included) was set as the limiting criteria for a site to be deemed suitable for visitation observation.

We studied visitation observation for 6 hr day^−1^ farmland^−1^ during the standardized peak visitation period. This effort was duplicated by another observer on each day in each farmland. Each observer visited four quadrats (within a farmland) within each hour. This was repeated across the 6 hr. In each quadrat, an observer spent 5 min for active observation followed by a 10‐min gap (within which the observer moved to the next sampling site). So within an hour, an observer actively sampled (4 quadrats × 5 min) = 20 min of visitation. This was repeated across 6 hr (6 min × 20 min = 120 min), and two observers (120 min × 2 = 240 min), and across 2 days (240 min × 2 min = 480 min). Hence, we observed 480 min/farmland across 16 quadrats (4 quadrat × 2 days × 2 observer) to understand the visitation pattern. These were the same 16 quadrats in which we had measured the number of flowers. We considered only those flower visitors (as pollinators) who made contact with the essential reproductive parts of the flowers, that is, the anther or stigma. This entire observation was repeated on the 2nd study year on the same farmlands.

#### 2.2.3 Data analysis

We plotted the rainfall data for both district and subdistrict across 24 years and checked for congruence. We fitted a linear model weighted for an unequal variance to examine the potential effect of rainfall on the average district level horticultural crop yield. We conducted pairwise tests to compare flower numbers, FFR, fruit quality, and yield between the two study years. We also compared the total and individual pollinator species visitation pattern across the farmlands between the study years using either paired *t* test or Wilcoxon signed‐rank test. In addition, we also performed generalized linear mixed models with farmland identity as random effects to compare flower numbers, fruit quality, and visitation pattern across farmlands over the two study years. As FFR was measured only once per farmland (number of flowers that converted to fruits out of 50 randomly tagged flowers), we used pairwise comparisons. For yield estimation per farmland, we also calculated the 95% confidence interval around the mean yield using the variation present in fruit weight and the number of flowers per farmland. We applied principal component analysis (PCA) to examine the dominant pollinators that were driving the pollinator community across the study years. We fitted generalized linear models with binomial error distribution to examine the relationship between FFR and pollinator visitation separately for both the years. Additionally, we calculated Shannon's diversity index of pollinators for each farmland across the study years. We also examined the impact of pollinator diversity on FFR, using a GLM with a quasibinomial error structure. We used R software (Team, 2018) for all our analyses (packages: ggplot2, sjPlot, vegan, car, glmmTMB, and lmtest).

## 3 RESULTS

### 3.1 Rainfall showed significant variation over the years in Bangalore urban district and Anekal

Our long‐term data revealed significant variation in the overall rainfall pattern in our study site over 24 years—Bangalore urban district: highest 1,290 mm, lowest 469 mm, and average 850 mm; Anekal: highest 1,343 mm, lowest 433 mm, and average 813 mm. Rainfall pattern across the district and taluk level showed a comparable trend with a high correlation coefficient of 0.89 (Pearson's correlation; *p* = 7.378e−09), indicating strong coherence (Figure 1e). We found the average yield of horticultural crops—including Chayote squash—increased with increasing rainfall (*R* ^2^ = .65, *F*_1, 4_ = 10.46, slope = 5.43, *p* = 003; Figure 1f), suggesting a significant influence of rainfall variation on crop yield. Earlier studies have also found such strong covariance between rainfall and crop yield and have reported that rainfall plays a critical role in crop production (Kang et al., 2009; Ray et al., 2015). However, we did not find a relationship between the crop yield of non‐pollinator‐dependent crop and rainfall (*F*_1,7_ = 0.38, *p* = .56) (Figure S3).

### 3.2 Pollinator visitation is essential for the reproduction of Chayote squash

Pollination exclusion experiment confirmed Chayote to be highly pollinator‐dependent. Pollinator‐restricted flowers (*N* = 79) bore no fruit, whereas open flowers (*N* = 80) bore 18 fruits (prop. test X^2^ = 8.0765, *df* = 1, *p* = .002; Figure S4), highlighting the importance of the pollinators.

### 3.3 Peak visitation time period of pollinators of Chayote squash

The primary pollinators of Chayote squash in our study area were four wild bee species: *Apis dorsata*, *Apis cerana*, *Apis florea*, and *Trigona* sp. As bees are not cultured in these districts, all these bee species depend critically on the surrounding natural habitat. Our preliminary observation revealed that the peak visitation duration for Chayote spanned between 7:30 a.m. and 2:30 p.m. (Figure S5).

### 3.4 Floral numbers and yield showed strong correspondence to rainfall shift

**Figure 2:**
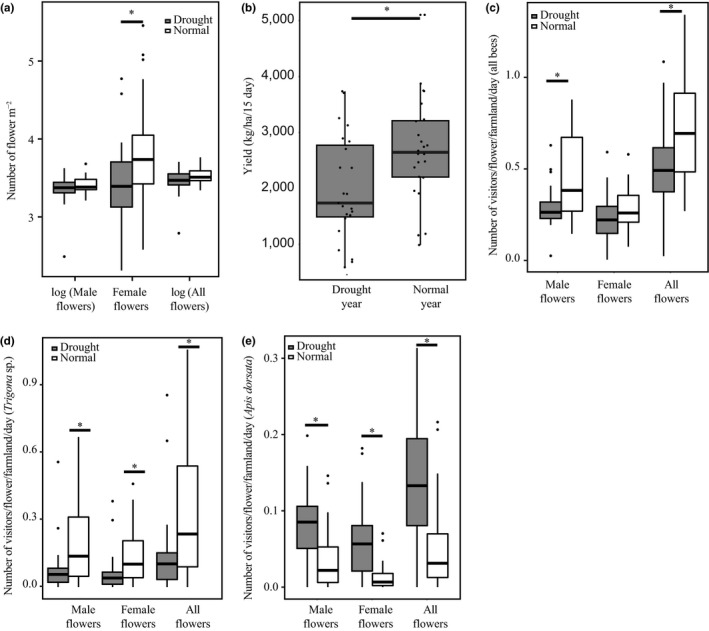
(a) Boxplot is showing a comparison of the log (number of total), female, and log (male flowers) found per square meter of the study farmlands (*N* = 24) between drought and normal years. The significant difference (female flowers) is highlighted using a “*” mark. The total number of flowers and male flowers is shown in log scale for better visualization of the distribution. (b) Boxplot showing the comparison of yield (number of fruits produced/farmland/15‐day harvest cycle) across 24 farmlands. (c)–(e) show the comparative visitation pattern of all bees, *Trigona* sp., and *Apis dorsata*, respectively, between drought and normal rainfall years

We found Chayote produced fewer number of female flowers during drought compared with normal rainfall year (paired *t* test female flower: *t* = 2.41, *df* = 23, *p* = .01; GLMM (Poisson) female flower: *p* = 0.006, Table S2). However, male flower production remained unaffected (Wilcoxon signed‐rank test male flower: *p* = .7, GLMM (Poisson) male flower: *p* = .91, paired *t* test all flower: *p* = .08; GLMM (Poisson) all flower: *p* = .15) (Figure 2a, Tables S3–S5). As female flowers lead to fruit formation, the decline in female flowers was expected to have a significant effect on plant yield. Interestingly, we did not find any effect of drought on FFR (paired *t* test FFR: *t* = 1.84, *df* = 22, *p* = .08), which is known to be a strong indicator of pollination service (Ne'Eman et al., 2010). We also did not find any impact of rainfall deficit on fruit quality (paired *t* test fruit weight: *p* = .4, length: *p* = .7, and width: *p* = .2; GLMM (Gaussian) fruit weight: *p* = .6, fruit length: *p* = .3, fruit width: *p* = .08) (Tables S6–S9, Figure S6). Our yield calculation revealed a decrease in the average yield of Chayote in the drought year (paired *t* test yield: *t* = −2.624, *df* = 22, *p* = .008, the difference in mean yield: 648.62 kg/hectare; Figure 2b; see Table S10 for the confidence interval of yield for each farmland). This decline in yield was concordant to the district level pattern of yield decline during decreasing rainfall (Figure 1f).

### 3.5 Pollinator communities showed drastic differences in composition between the two study years

Pollinator visitation (all pollinators combined) decreased in the drought year in comparison with the normal rainfall year (paired *t* test all flower: *t* = 2.6193, *df* = 23, *p* = .008; GLMM (Poisson) all flower: *p* = .0009; Figure 2c, Table S11). This was mainly driven by decreased visitation to male flowers (Wilcoxon signed‐rank test male flower: *p* = .002, GLMM (Poisson) male flower: *p* = .0004) (Tables S12 and S14). However, visitation of all bees to female flowers remained unchanged (paired *t* test female flowers: *p* = .22; GLMM (Poisson) female flower: *p* = .36) (Tables S12 and S15). We found that *Trigona* sp. visitation showed steep decline during drought across flower types (Wilcoxon signed‐rank test all flower: *V* = 244, *p* = .005, male flower: *V* = 254, *p* = .002, female flower: *V* = 234, *p* = .015; GLMM (Poisson) all flowers: *p* = 1.6 × 10^−6^, male flowers: *p* = 5.47 × 10^−5^, female flowers: *p* = .0072) (Figure 2d, Tables S16–S18). On the contrary, we found that *Apis dorsata* visitation increased significantly during drought, across all flower types (Wilcoxon signed‐rank test all flower: *V* = 35, *p* = .0004, male flower: *V* = 39, *p* = .0008, female flower: *V* = 29, *p* = .002; GLMM (Poisson) all flower: *p* = .0002, male flower: *p* = .015, female flower: *p* = .004; Figure 2e, Tables S19–S21). Visitation pattern of *Apis cerana* was comparable to the overall visitation trend (Wilcoxon signed‐rank test all flowers: *p* = .009, male flowers: *p* = .002, female flowers: *p* = .14; GLMM (Poisson) all flowers: *p* = 1.97 × 10^−5^, male flowers: *p* = 4.45 × 10^−5^, female flowers: *p* = .06) (Figure S7a, Tables S13A and S22–S24), while *Apis florea* visitation remained unperturbed during rainfall variation (Wilcoxon signed‐rank test all flower: *p* = .7, male flower: *p* = .9, female flower: *p* = .6; GLMM (Poisson) all flowers: *p* = .41, male flowers: *p* = .56, female flowers: *p* = .43) (Figure S7b, Tables S13B and S25–S27). These changes in pollinator visitation during rainfall variation led to a drastic shift in the pollinator community composition across most farmlands (Figure S8).

### 3.6 What maintains the pollination service despite pollinator community shift during rainfall variation: Ecosystem stabilizing mechanism

**Figure 3:**
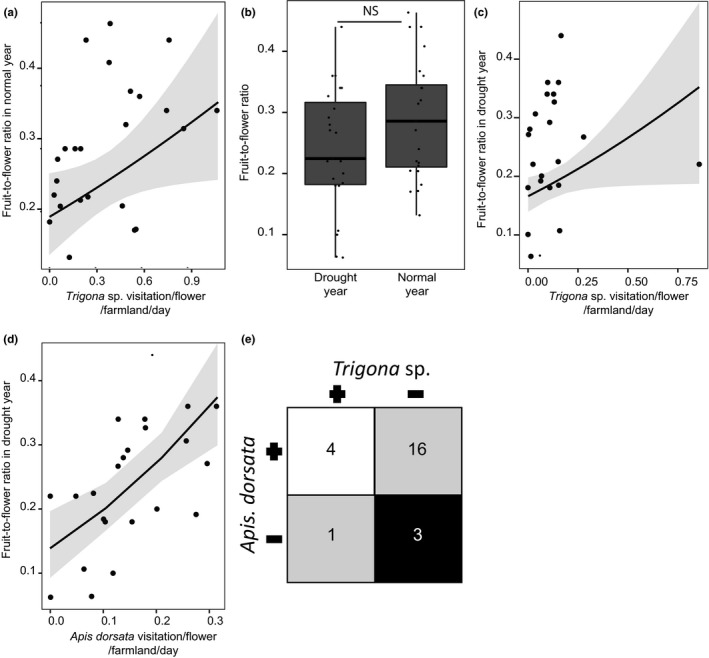
(a) Scatter plot showing the relationship of *Trigona* sp. visitation and fruit‐to‐flower ratio (FFR) across 24 farmlands during normal rainfall year. The gray band indicates the 95% confidence interval. (b) Boxplot showing the comparison of FFR between the normal and the drought years [NS: not significant]. (c) and (d) Scatter plots showing the relationships of *Trigona* sp. and *Apis dorsata* visitation and FFR during a drought year. (e) Matrix is showing that 16 out of 24 farmlands had declined in *Trigona* sp. visitation during the drought with a concomitant increase in *Apis dorsata* visitation

We examined which pollinators affected FFR during the normal rainfall year as FFR is a strong predictor of pollination service. We did not find an effect of pollinator diversity on FFR (Table S28B). However, we found that *Trigona* sp. was the sole pollinator that strongly covaried and positively influenced FFR (*p* = .03; Figure 3a, Table S29A). We found this result surprising, as, during drought, despite a drastic decline in *Trigona* sp. visitation (Figure 2d), the FFR remained unperturbed (Figure 3b). We applied the concept of ecosystem stabilizing mechanism to understand the underlying processes that might have attributed to unchanged FFR during drought despite a drastic decline in the crucial pollinator. Ecosystem stabilizing mechanisms suggest that in scenarios of diversity loss, an abundance of one species providing service increases during a decrease in the abundance of other species. This is referred to as “Density compensation” (Winfree & Kremen, 2009). We hypothesized that during the drought when the key pollinator declined, “density compensation” maintained the FFR despite the loss of critical pollinator of Chayote.

We found that *Trigona* sp. was a weak driver of FFR during the drought year (*p* = .046, Figure 3c, Table S29B). However, *Apis dorsata* visitation strongly covaried and influenced FFR (*p* = 9.06 × 10^−5^, Figure 3d, Table S29B) during drought and was critical in maintaining the FFR comparable to normal monsoon year (also see Figure S9 and Table S30 for models excluding the extremely high *Trigona* sp. data point). Concordant to normal rainfall year, we did not find a relationship between the diversity of the pollinators and FFR during the drought year (Table S28A). We also established a typical scenario of “density compensation,” at the individual farm level, by showing that the decline in *Trigona* sp. was spatially coupled with an increase in *A. dorsata*. We developed a pairwise matrix to examine how many farmlands showed a decrease in *Trigona* sp. during the drought with a concomitant increase in *A. dorsata* and vice versa. For this analysis, we marked any decline in bee visitation during the drought (in comparison with the normal year) as negative (−ve) and any increase as positive (+ve). In this way, we scored each farmland. We developed such visitation difference arrays for each of *A. dorsata* and *Trigona* sp. using the absolute value of the difference in bee visitation (normal—drought). We then developed a pairwise interaction matrix of the two bee types based on their response toward drought. We assigned farmlands to “−−” quadrant where both bee types decreased due to drought (Figure 3e). Similarly, we assigned each farmland to the other three quadrants (“++,” “+−,” and “−+”) based on the pairwise interaction between the two bee types (Figure 3e). We expected that most farmlands would exhibit a decline in *Trigona* sp. visitation with a concomitant increase in *A. dorsata* visitation during drought (i.e., +− quadrant) (Figure 3e). We performed pairwise proportion test to compare the proportion of farmlands in each quadrant. Additionally, we also performed Wilcoxon signed‐rank test on each farmland to compare whether the increase in *A. dorsata* visitation (1‐tailed test with alternative hypothesis set as greater) and decrease in *Trigona* sp. visitation (1‐tailed test with alternative hypothesis set as lesser) were significant between drought and normal rainfall years.

Most numbers of farmlands (*n* = 16 out of 24, 67%) landed on the quadrant where *Trigona* sp. visitation declined with concomitant increase in *A. dorsata* visitation during drought (i.e., “−+” quadrant, Paired prop. test with FDR correction, quadrant −+ vs. −−, ++, +−: *p* = .001, .002, .0001) (Figure 3e). All the other quadrants had comparable numbers of farmlands (Figure 3e). This result supported our hypothesis that across the majority of the farmlands, the decline in *Trigona* sp. visitation during the drought was compensated by an increase in *A. dorsata* visitation. Our Wilcoxon signed‐rank test revealed that 19 (79%) (Table S31) farmlands showed a significant increase in *A. dorsata* visitation during drought. We also found that 18 farmlands (75%) showed a significant decline in *Trigona* sp. visitation during drought (Table S31). 14 (58%) farmlands showed a significant decrease in *Trigona* sp. visitation with a concomitant increase in *A. dorsata* visitation (marked in bold in Table S31).

## 4 DISCUSSION

Global climate change scenario has been predicted to make the climatic extremities pronounced and erratic (Cai et al., 2014; Karl & Trenberth, 2003; Thornton et al., 2014; Vasseur et al., 2014). In this study, we examined how the yield of pollinator‐dependent horticultural crops—in the agrarian hub of a developing nation—responds to rainfall fluctuations. We showed that the rainfall fluctuation was significant over the last three decades at both local and regional landscapes. We also established that this fluctuation had a strong correspondence with yield fluctuation of horticultural crops in this area. Studies have generally found a critical impact of rainfall decline on plant components leading to a drop in yield (Alqudah et al., 2011; Mendelsohn, 2008; Thomson, 2016). Concordant to these studies, our study also revealed a substantial drop in the number of female flowers produced in the dominant horticultural crop (Chayote squash) during drought, which in turn had a significant impact on the yield. We attribute this mainly to lack of rainfall due to the following reasons: (a) We did not find any significant shift in the agricultural practices between the study years and (b) the study been done for two consecutive years reduced the possibility of any other long‐term abiotic changes to play a crucial role. However, following the trend revealed in our regional level analysis (Figure 1f), we expected the yield decline to be more severe in the study crop (Chayote).

Drought tolerance in plants, although been examined thoroughly since the advent of agriculture, is taking precedence in the current climatic scenario. It has generally been attributed to the plant traits that provide such tolerance during climatic unpredictability. However, such tolerance capability—often measured as unperturbed yield in crops—can also critically depend on pollinator's tolerance capacity, in pollinator‐dependent crops such as Chayote. The yield can critically decline if the pollinators are sensitive to climatic shift. Rainfall decline has been shown to cause significant shifts in pollinator composition, visitation pattern, and sometimes local extinction (Devoto et al., 2005; Harrison, 2000, 2001). Concordant to these, we also found a substantial decline in the vital pollinators' visitation during drought (*Trigona* sp.). Studies have shown that *Trigona* sp. is sensitive to drought‐like events that cause fluctuation in resource availability. Inoue et al. (1993) showed that *El Nino* seasons with less rainfall could affect the colony foundation in *Trigona* sp.

One of the key indicators of pollination efficiency is FFR (Ne'Eman et al., 2010). Under declining pollinator visitation, it is expected to show a significant impact (Ne'Eman et al., 2010). Interestingly in our study, we did not find any shift in FFR despite a steep drop in rainfall. This was surprising as the *Trigona* sp.—the key pollinator whose visitation during normal rainfall year explained the variation in FFR—showed the steepest decline among all the pollinators of Chayote. We summarized that the drought‐induced decline in the critical pollinator's abundance seemed to have little impact on pollination efficiency (FFR). Further analysis of the pollinator visitation profile revealed that this was achieved through ecosystem stabilizing mechanism called “density compensation.”

“Density or Numerical compensation” is a phenomenon where in the event of rapid diversity loss, one species compensates for the functional loss of another (Winfree & Kremen, 2009). During drought, *Apis dorsata* visitation increased and positively affected the FFR. We showed that this compensated for the functional loss of *Trigona* sp. across the majority (67%) of the farmlands. It is known that *Trigona* sp. can recruit foragers promptly in response to a sudden increase in resource availability, such as synchronous flowering in agricultural lands (Nagamitsu & Inoue, 2002). This capability makes them ideal pollinator of the agricultural landscape. However, their limited foraging range makes them vulnerable to resource scarcity, during climatic anomaly like drought (Inoue et al., 1993; Nagamitsu & Inoue, 2002). On the contrary, *Apis dorsata*, a long distance forager (Koeniger & Koeniger, 1980), can thrive on fluctuating resources (Itioka et al., 2001; Momose & Karim, 2005), making them a robust pollinator despite climatic unpredictability. This robustness added with lack of potential competitors (Koeniger & Vorwohl, 1979) likely enabled *Apis dorsata* to compensate for the lack of *Trigona* sp. during the drought year.

## 5 POTENTIAL IMPLICATIONS

### 5.1 Importance of understanding core ecological ideas such as ecosystem stabilization

Winfree and Kremen (2009) had hypothesized that density compensation should be less frequent in human‐disturbed landscapes owing to its associations with resource availability over the spatial scale. However, our study shows that even in human‐disturbed landscapes, it can occur over the temporal domain. Density compensation phenomenon might be less observed in temperate as well as new world tropics because solitary bees are more abundant in these regions compared with social ones (Roubik, 2005). Studies have shown that social bees are more adapted to fluctuating resources as they can either migrate and forage over large distances, or alter the colony size based on resource availability (Momose & Karim, 2005). Hence, in the old world tropical country like India, where social bee diversity is significantly higher, the probability of such a phenomenon is considerably greater.

### 5.2 Understanding the importance of surrounding habitat quality for pollinator sustenance

Our study—using paired year cropping data during climatic fluctuation—emphasizes the role of natural bee diversity as an insurance mechanism for a dominant pollinator‐dependent crop in the dearth of critical pollinators (Kremen, Williams, & Thorp, 2002). It also demonstrates that this diversity aids in sustaining the agricultural yield during the potential stress created by sudden climatic shift. We speculate that for sustainable pollination service across the production landscape, the surrounding areas (semi‐wild as well as protected areas) might play a critical role. Though this was beyond the scope of the present study, it will be important to examine the role of surrounding landscape factors on pollinator visitation.

### 5.3 Importance of climatic instability proof sustainable models of agriculture

Sustainable agriculture models in human‐dominated habitat often work under the assumption of the constant favorable environment. Human‐induced global warming has made climatic conditions unpredictable and more extreme (Cai et al., 2014; Thornton et al., 2014; Vasseur et al., 2014). Such climatic changes can be related to the significant decline in crop yield (Abewoy, 2018; Ray et al., 2015), as was also evident in our study for all horticultural crops (Figure 1f). Hence, future models should encompass for such variation to be viable at longer timescales. In our study, we found that *Trigona* sp. was the critical pollinator for Chayote squash in normal rainfall year. However, only through our observations during a drought year, we found that an alternate bee species (*Apis dorsata*) was critical in sustaining the pollination service during drought. Hence, any sustainable agricultural model that accounted for only *Trigona* sp. as the important pollinator would have failed under this climatic uncertainty. We suggest that more studies in future should examine pollination service (and other essential ecosystem services) under variable climatic stressors to develop a genuinely robust sustainable agricultural model in human‐dominated landscapes.

## CONFLICT OF INTEREST

The authors declare no conflict of interest.

## AUTHOR CONTRIBUTIONS

RM conceived the study; acquired funding; conducted field surveys, designed and executed field experiments and observations, collected ground‐truth points; collected secondary data, digitized and analyzed the data; conducted statistical analysis, prepared figures, and wrote the manuscript. RD carried out statistical analysis, prepared figures, and gave critical comments on the manuscript. SD conceived the study; facilitated in fundraising; and gave critical comments on the manuscript.

## Data Availability

All data have been made available in Dryad (https://doi.org/10.5061/dryad.0n5v168).
