# Supplementary material for: Diversity matters: Effects of density compensation in pollination service during rainfall shift
Source: Ecol Evol. 2019 Aug 18;9(17):9701–11. doi: 10.1002/ece3.5500 (PMC6745652; doi:10.1002/ece3.5500)
Supplement: Supplementary file 1 [file ECE3-9-9701-s001.docx]

# **Figures**


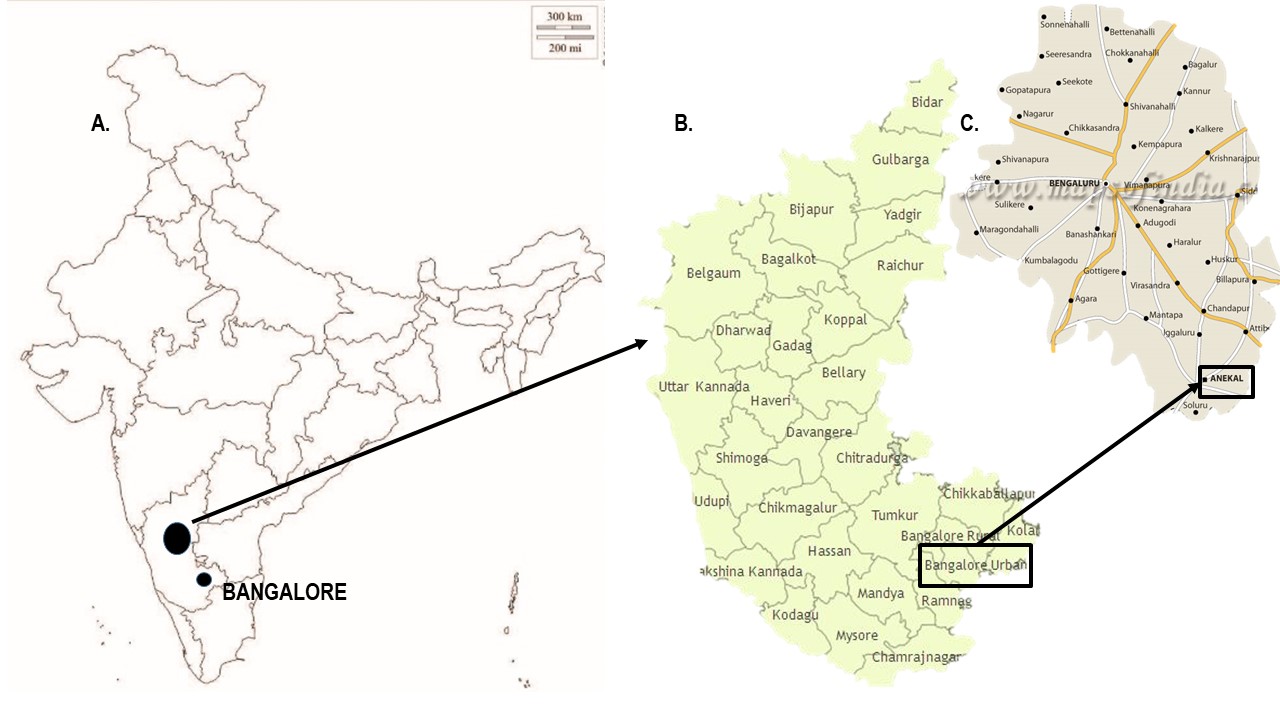
**Figure S1: Figure showing the location of our study Anekal A) Map of India with the state Karnataka and Bangalore city marked. B) Inset showing Bangalore urban district within Karnataka. C) Anekal sub-district within the Bangalore urban-district map (Map source: mapsofindia.com)**

**Figure S2: Figure depicting the sampling design for visitation observation in a farmland. Each diagonal was used by a different observer. The sampling quadrats were chosen randomly. This sampling design was repeated on the second day of visitation observation when four new random quadrats/observer were chosen.**

**
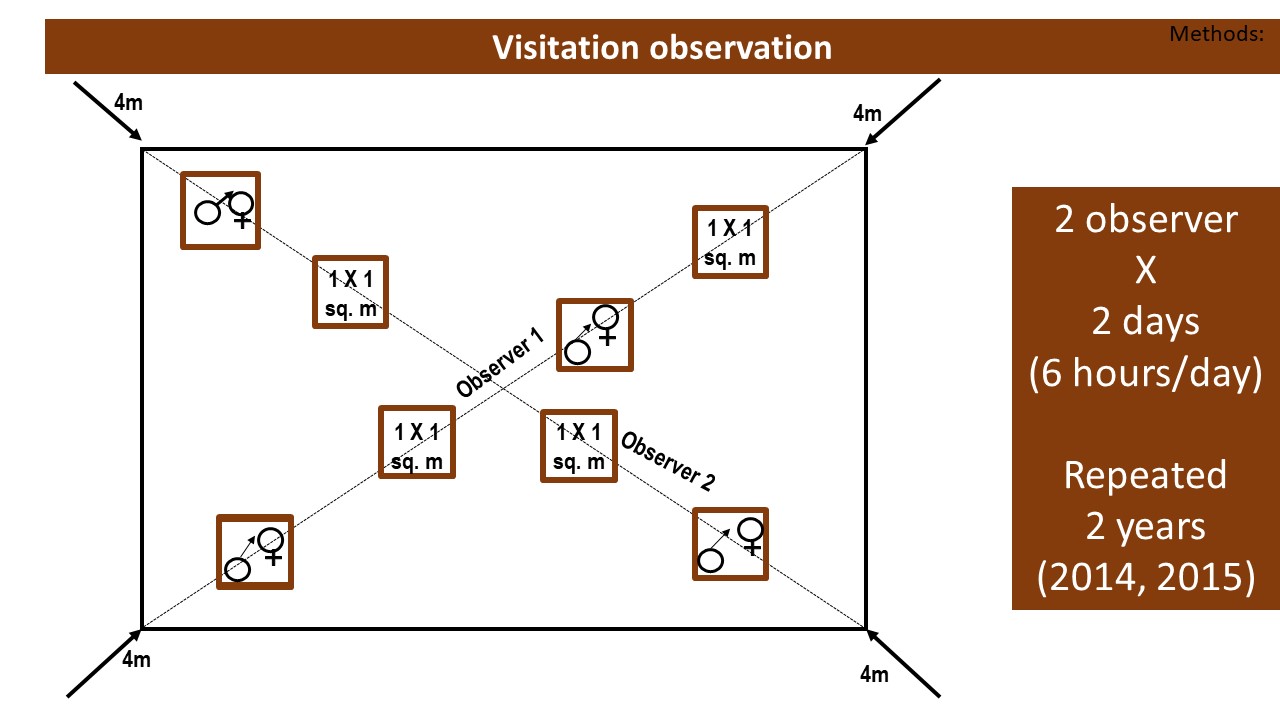
**

**Figure S3: Regression plot is showing the dependence of crop yield (Y-axis) on rainfall (X-axis). The crops whose yields were used to calculate the average yield were; Paddy, Maize, Finger millet. The crop composition remained constant across the years.**


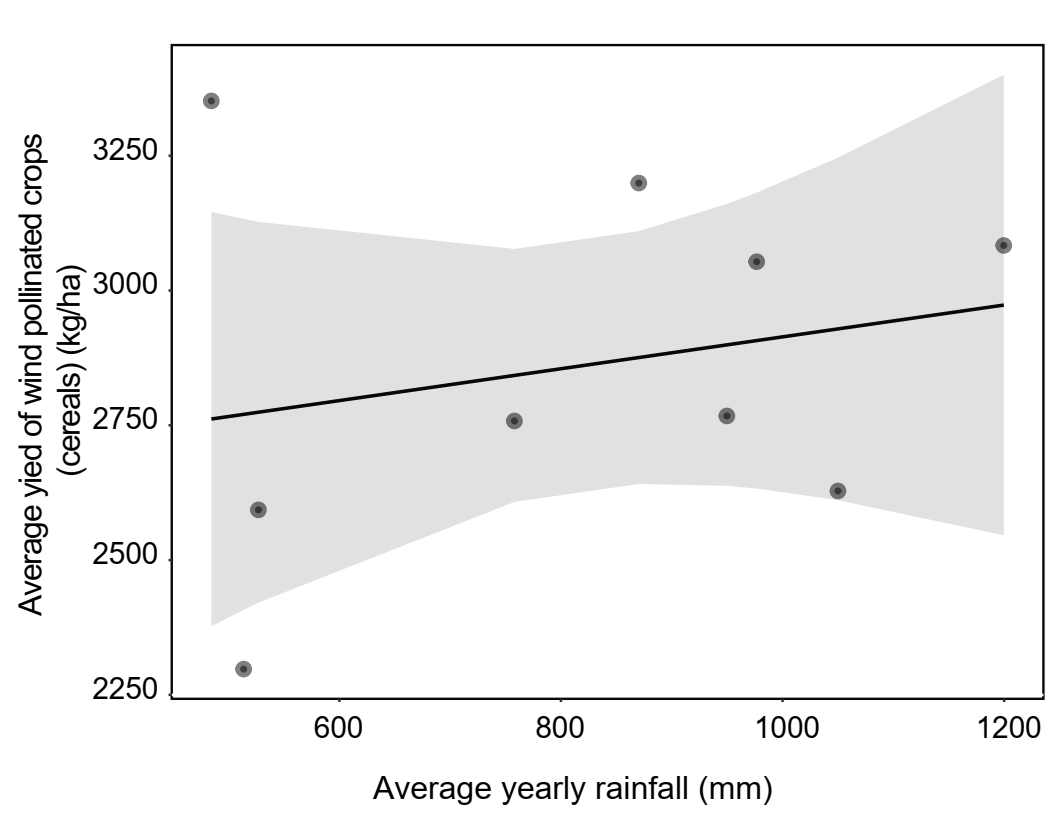


**Figure S4: Bar plot showing the result of pollinator exclusion experiment. Open: Control treatment with tagged female buds. Pollinator-exclusion: Bagged flowers with no pollinator access.**

**Figure S5: Bar plot showing pollinator visitation timing across the day with peak visitation hours. The error bars represent standard errors calculated from the visitation pattern across three farmlands.**

**Figure S6: Comparison of average fruit weight, length and width between drought and the normal rainfall year. The y-axis represents the log value of fruit weight (in gm) and fruit length and width (in cm).**


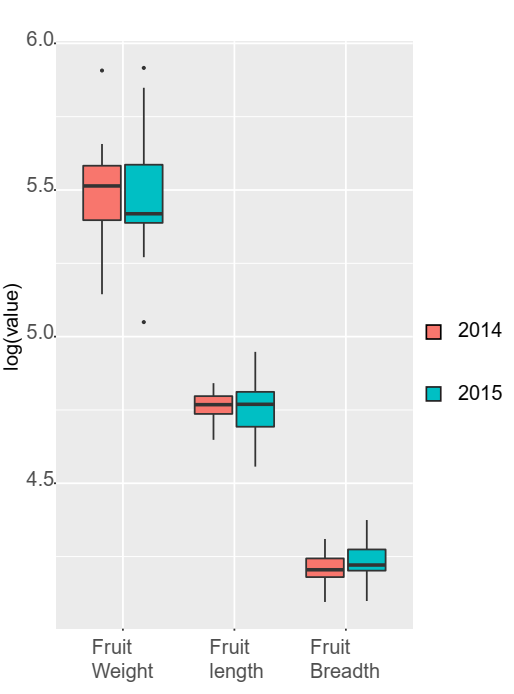


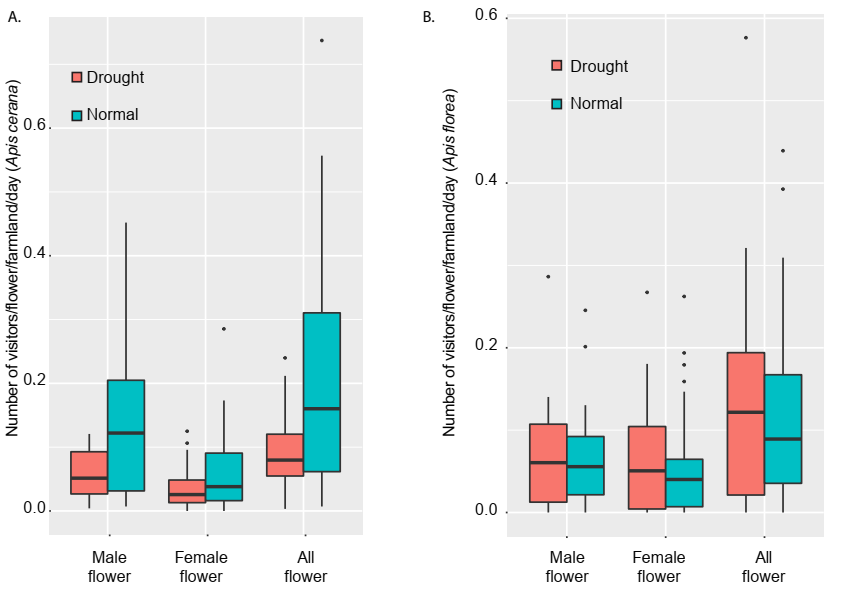
**Figure S7: Comparison of average A. *Apis cerana* B. *Apis florea* visitation pattern between normal and drought year.**

**Figure S8: Principal component analysis of the pollinator communities across the drought and the normal rainfall year showing a significant difference in composition. The red dots represent the pollinator community of each farmland during drought. The blue dots represent the pollinator community of the same farmlands during normal rainfall year.**


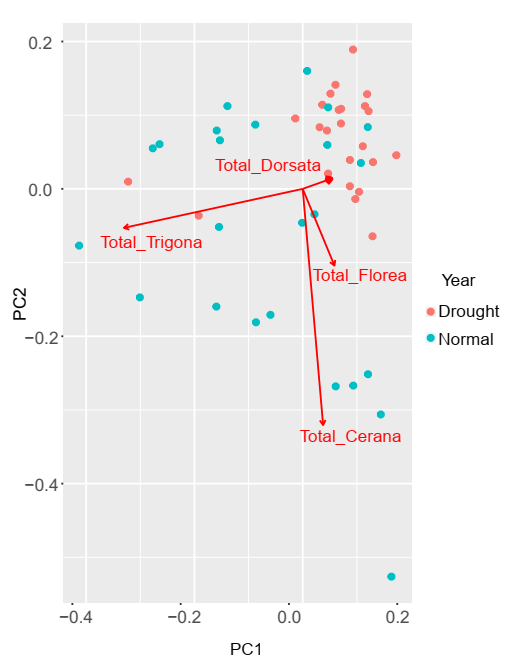


**Figure S9: Relationship between bee visitation and FFR during drought year, after removing the farmland with very high *Trigona* sp. visitation (0.85). A) Relationship between FFR and *A. dorsata* visitation under varying proportion of *A. florea* visitation. B) Relationship between FFR and *Trigona* sp. visitation under varying proportion of *A. florea* visitation. C) Frequency distribution of *A. florea* visitation/flower/farmland/day. Both *A. dorsata* and *Trigona* sp. visitation positively affected FFR when *A. florea* visitation was low (red lines) or medium (blue lines). However, with increase in *A. florea* visitation (>=0.27) positive effect of *A. dorsata* and *Trigona* sp. on FFR declined (green lines). However it should be noted that only few farmlands (4) had high (>=0.27) *A. florea* visitation (panel C).**


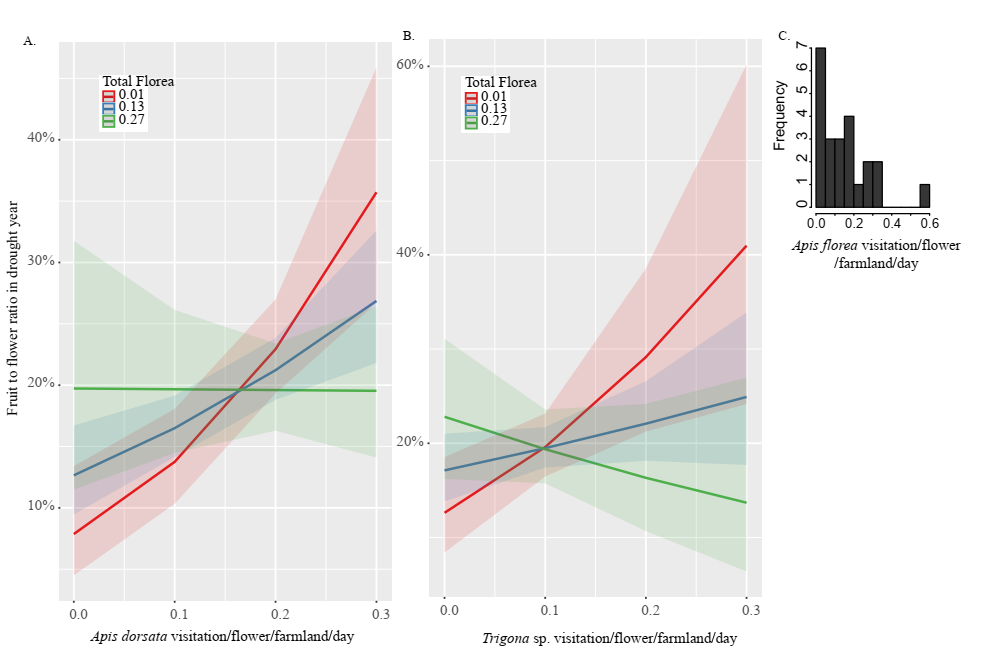


**Tables**

**Table S1: Farmland visitation sampling dates for year 2014 and 2015.**

| **Farm id** | **2014** | **2015** |
| --- | --- | --- |
| Field1 | 03-11-2014 | 12-10-2015 |
| Field11 | 08-09-2014 | 05-08-2015 |
| Field14 | 09-08-2014 | 07-09-2015 |
| Field17 | 14-10-2014 | 20-09-2015 |
| Field19 | 10-10-2014 | 10-09-2015 |
| Field2 | 09-07-2014 | 06-08-2015 |
| Field22 | 01-10-2014 | 03-10-2015 |
| Field25 | 02-11-2014 | 24-10-2015 |
| Field28 | 04-10-2014 | 09-10-2015 |
| Field30 | 11-09-2014 | 11-08-2015 |
| Field4 | 25-06-2014 | 06-07-2015 |
| Field6 | 18-10-2014 | 15-09-2015 |
| Field7 | 25-08-2014 | 15-08-2015 |
| FieldA | 28-08-2014 | 08-08-2015 |
| FieldF | 25-09-2014 | 27-08-2015 |
| FieldF33 | 21-10-2014 | 11-10-2015 |
| FieldK | 24-10-2014 | 14-10-2015 |
| FieldL | 28-10-2014 | 16-10-2015 |
| FieldM | 30-10-2014 | 18-10-2015 |
| FieldN2 | 21-08-2014 | 07-09-2015 |
| FieldO | 15-11-2014 | 03-11-2015 |
| FieldP11 | 13-11-2014 | 28-10-2015 |
| FieldSN14 | 16-10-2014 | 27-09-2015 |
| FieldZ | 07-10-2014 | 29-09-2015 |

**Table S2: Comparison of number of female flowers across the normal and the drought year with farm id as a random factor**

| Poisson family |  |  |  |  |
| --- | --- | --- | --- | --- |
| AIC | BIC | log likelihood | Deviance | Residual_Df |
| 2934.5 | 2948.4 | -1464.2 | 2928.5 | 768 |
|  |  |  |  |  |
| Random effects: |  |  |  |  |
|  |  |  |  |  |
| Conditional model: |  |  |  |  |
| Groups | Name | Variance | Standard deviation |  |
| Farm_ID | Intercept | 0.0074 | 0.086 |  |
|  |  |  |  |  |
| Number of observation | 771 | Groups: | 24 |  |
|  |  |  |  |  |
|  |  |  |  |  |
|  |  |  |  |  |
| Conditional model: |  |  |  |  |
|  | Estimate | Std.error | z-value | P |
| Intercept | 1.25 | 0.033 | 38.09 | 2X10^-16^ |
| Female flower Normal year(2015) | 0.1 | 0.038 | 2.72 | 0.006 |
|  |  |  |  |  |

**Table S3: Comparison of plant investment in the form of the number of male and total flowers (all flowers) produced during drought vs normal rainfall year**

|  | **Test** | **Statistic** | **DF** | **P-value** |
| --- | --- | --- | --- | --- |
| Total flower number (All flowers) | Paired t-test | t=1.38 | 23 | 0.08 |
| Male flower number | Wilcoxon signed rank test | Z= -0.62898 |  | 0.74 |

**Table S4: Comparison of number of male flowers across the normal and the drought year with farm id as a random factor**

|  |  |  |  |  |
| --- | --- | --- | --- | --- |
| Poisson family |  |  |  |  |
| AIC | BIC | log likelihood | Deviance | Residual_Df |
| 5713.8 | 5727.9 | -2853.9 | 5707.8 | 793 |
|  |  |  |  |  |
| Random effects: |  |  |  |  |
|  |  |  |  |  |
| Conditional model: |  |  |  |  |
| Groups | Name | Variance | Standard deviation |  |
| Farm_ID | Intercept | 0.005 | 0.071 |  |
|  |  |  |  |  |
| Number of observation | 796 | Groups: | 24 |  |
|  |  |  |  |  |
|  |  |  |  |  |
|  |  |  |  |  |
| Conditional model: |  |  |  |  |
|  | Estimate | Std.error | z-value | P |
| Intercept | 3.45 | 0.017 | 201.95 | 2X10^-16^ |
| Male flower Normal year(2015) | 0.001 | 0.013 | 0.11 | 0.91 |
|  |  |  |  |  |

**Table S5: Comparison of number of all flowers across the normal and the drought year with farm id as a random factor**

| Poisson family |  |  |  |  |
| --- | --- | --- | --- | --- |
| AIC | BIC | log likelihood | Deviance | Residual_Df |
| 5772.2 | 5786.2 | -2883.1 | 5766.2 | 793 |
|  |  |  |  |  |
| Random effects: |  |  |  |  |
|  |  |  |  |  |
| Conditional model: |  |  |  |  |
| Groups | Name | Variance | Standard deviation |  |
| Farm_ID | Intercept | 0.005 | 0.07 |  |
|  |  |  |  |  |
| Number of observation | 796 | Groups: | 24 |  |
|  |  |  |  |  |
|  |  |  |  |  |
|  |  |  |  |  |
| Conditional model: |  |  |  |  |
|  | Estimate | Std.error | z-value | P |
| Intercept | 3.55 | 0.017 | 214.7 | 2X10^-16^ |
| All flower Normal year(2015) | 0.018 | 0.012 | 1.46 | 0.15 |

**Table S6: Comparison of fruit weight, length, and width between the drought and the normal rainfall year.**

|  | **Test** | **Statistic** | **DF** | **P-value** |
| --- | --- | --- | --- | --- |
| Fruit weight | Paired t-test | t= 0.2 | 22 | 0.42 |
| Fruit length | Paired t-test | t= -0.5 | 19 | 0.7 |
| Fruit width | Paired t-test | t= 0.9 | 19 | 0.2 |

**Table S7: Comparison of fruit weight between the drought and the normal rainfall year with farm id as a random factor.**

| Gaussian family |  |  |  |  |
| --- | --- | --- | --- | --- |
| AIC | BIC | log likelihood | Deviance | Residual_Df |
| 4978 | 4994.6 | -2485 | 4970 | 456 |
|  |  |  |  |  |
| Random effects: |  |  |  |  |
|  |  |  |  |  |
| Conditional model: |  |  |  |  |
| Groups | Name | Variance | Standard deviation |  |
| Farm_ID | Intercept | 1255 | 35.42 |  |
| Residual |  | 2560 | 50.59 |  |
| Number of observation | 460 | Groups: | 23 |  |
|  |  |  |  |  |
| Dispersion estimate: | 2.56X10^3^ |  |  |  |
|  |  |  |  |  |
| Conditional model: |  |  |  |  |
|  | Estimate | Std.error | z-value | P |
| Intercept | 245.13 | 8.11 | 30.25 | 2X10^-16^ |
| Fruit weight Normal year(2015) | 2.23 | 4.72 | 0.47 | 0.64 |
|  |  |  |  |  |

**Table S8: Comparison of fruit length between the drought and the normal rainfall year with farm id as a random factor.**

| Gaussian family |  |  |  |  |
| --- | --- | --- | --- | --- |
| AIC | BIC | log likelihood | Deviance | Residual_Df |
| 3481 | 3497.5 | -1736.5 | 3473 | 456 |
|  |  |  |  |  |
| Random effects: |  |  |  |  |
|  |  |  |  |  |
| Conditional model: |  |  |  |  |
| Groups | Name | Variance | Standard deviation |  |
| Farm_ID | Intercept | 58.22 | 7.63 |  |
| Residual |  | 97.93 | 9.89 |  |
| Number of observation | 460 | Groups: | 23 |  |
|  |  |  |  |  |
| Dispersion estimate: | 97.9 |  |  |  |
|  |  |  |  |  |
| Conditional model: |  |  |  |  |
|  | Estimate | Std.error | z-value | P |
| Intercept | 118.28 | 1.72 | 68.79 | 2X10^-16^ |
| Fruit Length Normal year(2015) | -1.04 | 0.92 | -1.13 | 0.258 |

**Table S9: Comparison of fruit width between the drought and the normal rainfall year with farm id as a random factor.**

| Gaussian family |  |  |  |  |
| --- | --- | --- | --- | --- |
| AIC | BIC | log likelihood | Deviance | Residual_Df |
| 2983.7 | 3000.2 | -1487.8 | 2975.7 | 456 |
|  |  |  |  |  |
| Random effects: |  |  |  |  |
|  |  |  |  |  |
| Conditional model: |  |  |  |  |
| Groups | Name | Variance | Standard deviation |  |
| Farm_ID | Intercept | 11.05 | 3.323 |  |
| Residual |  | 34.14 | 5.84 |  |
| Number of observation | 460 | Groups: | 23 |  |
|  |  |  |  |  |
| Dispersion estimate: | 34.1 |  |  |  |
|  |  |  |  |  |
| Conditional model: |  |  |  |  |
|  | Estimate | Std.error | z-value | P |
| Intercept | 68.36 | 0.79 | 86.19 | 2X10^-16^ |
| Fruit Width Normal year(2015) | 0.96 | 0.544 | 1.76 | 0.08 |

**Table S10: Showing the confidence interval for yield for each farm across the drought and the normal rainfall year. Please note yield of farm N2 is missing in 2014.**

| Farm ID | Yield_14_5CI | Yield_14 | Yield_14_95CI | Yield_15_5CI | Yield_15 | Yield_15_95CI |
| --- | --- | --- | --- | --- | --- | --- |
| Field1 | 1456.45 | 1907.05 | 2384.30 | 4357.20 | 5101.94 | 5902.95 |
| Field11 | 2230.83 | 2704.62 | 3205.29 | 1997.72 | 2467.53 | 2985.11 |
| Field14 | 1940.94 | 2367.16 | 2826.87 | 1963.95 | 2614.57 | 3342.71 |
| Field17 | 3152.59 | 3735.82 | 4359.33 | 2129.39 | 2952.23 | 3908.86 |
| Field19 | 2978.10 | 3706.56 | 4500.44 | 2188.38 | 2743.29 | 3359.24 |
| Field2 | 509.16 | 590.92 | 769.98 | 1899.78 | 2192.06 | 2504.56 |
| Field22 | 2648.18 | 3255.46 | 3913.19 | 2206.10 | 2768.85 | 3374.38 |
| Field25 | 1345.24 | 1538.00 | 1743.20 | 993.13 | 1188.68 | 1400.49 |
| Field28 | 2694.15 | 3126.85 | 3585.70 | 2794.87 | 3232.17 | 3694.25 |
| Field30 | 2342.78 | 2893.60 | 3495.43 | 2279.51 | 2616.25 | 2970.67 |
| Field4 | 1190.83 | 1586.59 | 2004.34 | 2516.96 | 3202.13 | 3969.55 |
| Field6 | 1609.97 | 1902.03 | 2216.52 | 3208.73 | 3872.70 | 4568.43 |
| Field7 | 555.33 | 728.94 | 923.67 | 1533.34 | 1955.81 | 2428.79 |
| FieldA | 1263.69 | 1518.87 | 1797.30 | 1608.07 | 1910.11 | 2237.20 |
| FieldF | 1393.70 | 1680.75 | 1991.24 | 836.31 | 1162.62 | 1541.82 |
| FieldF33 | 562.26 | 686.09 | 821.55 | 2049.55 | 2481.15 | 2950.90 |
| FieldK | 1030.00 | 1238.44 | 1463.03 | 1876.16 | 2206.59 | 2562.48 |
| FieldL | 1217.15 | 1637.46 | 2120.00 | 1940.81 | 2374.97 | 2852.90 |
| FieldM | 1239.57 | 1461.95 | 1701.88 | 3146.07 | 3747.31 | 4390.44 |
| FieldN2 |  |  |  | 2822.39 | 3515.29 | 4266.46 |
| FieldO | 1524.08 | 1738.13 | 1964.43 | 2302.28 | 2673.31 | 3066.74 |
| FieldP11 | 730.51 | 893.96 | 1073.12 | 2451.65 | 2836.25 | 3246.32 |
| FieldSN14 | 1912.39 | 2370.55 | 2876.70 | 847.04 | 987.51 | 1138.72 |
| FieldZ | 2382.19 | 2838.98 | 3333.99 | 3014.33 | 3739.07 | 4540.93 |

| Poisson family |  |  |  |  |
| --- | --- | --- | --- | --- |
| AIC | BIC | log likelihood | Deviance | Residual_Df |
| 1370 | 1383.9 | -682 | 1364 | 768 |
|  |  |  |  |  |
| Random effects: |  |  |  |  |
|  |  |  |  |  |
| Conditional model: |  |  |  |  |
| Groups | Name | Variance | Standard deviation |  |
| Farm_ID | Intercept | 0.056 | 0.24 |  |
|  |  |  |  |  |
| Number of observation | 771 | Groups: | 24 |  |
|  |  |  |  |  |
|  |  |  |  |  |
|  |  |  |  |  |
| Conditional model: |  |  |  |  |
|  | Estimate | Std.error | z-value | P |
| Intercept | -0.63 | 0.09 | -7.4 | 1.87X10^-13^ |
| All bee visit all flower  Normal year(2015) | 0.3 | 0.092 | 3.31 | 0.0009 |

**Table S11: Comparison of all bee visitation to all the flowers between the drought and the normal rainfall year with farm id as a random factor.**

**Table S12: Comparison of visitation between the drought and the normal rainfall year for all pollinators**

|  | **Test** | **Statistic** | **DF** | **P-value** |
| --- | --- | --- | --- | --- |
|  |  |  |  |  |
| All pollinators visiting male flowers | Wilcoxon signed rank test | V=253 |  | 0.0022 |
| All pollinators visiting female flowers | Paired t-test | T=1.26 | 23 | 0.22 |

**Table S13: Comparison of visitation between the drought and the normal rainfall year for A) *Apis cerana*, and B) *Apis florea***

| A) |  |  |  |  |
| --- | --- | --- | --- | --- |
| *Apis cerana* visiting the total number of flowers | Wilcoxon signed rank test | V=239 |  | 0.009 |
| *Apis cerana* visiting male flowers | Wilcoxon signed rank test | V=253 |  | 0.002 |
| *Apis cerana* visiting female flowers | Wilcoxon signed rank test | V= 203 |  | 0.14 |
| B) |  |  |  |  |
| *Apis florea* visiting the total number of flowers | Wilcoxon signed rank test | V=136 |  | 0.70 |
| *Apis florea* visiting male flowers | Wilcoxon signed rank test | V=145 |  | 0.90 |
| *Apis florea* visiting female flowers | Wilcoxon signed rank test | V=122 |  | 0.64 |

**Table S14: Comparison of all bee visitation to all the male flowers between the drought and the normal rainfall year with farm id as a random factor.**

| Poisson family |  |  |  |  |
| --- | --- | --- | --- | --- |
| AIC | BIC | log likelihood | Deviance | Residual_Df |
| 1040.4 | 1054.5 | -517.2 | 1034.4 | 793 |
|  |  |  |  |  |
| Random effects: |  |  |  |  |
|  |  |  |  |  |
| Conditional model: |  |  |  |  |
| Groups | Name | Variance | Standard deviation |  |
| Farm_ID | Intercept | 0.056 | 0.24 |  |
|  |  |  |  |  |
| Number of observation | 796 | Groups: | 24 |  |
|  |  |  |  |  |
|  |  |  |  |  |
|  |  |  |  |  |
| Conditional model: |  |  |  |  |
|  | Estimate | Std.error | z-value | P |
| Intercept | -1.22 | 0.1 | -11.73 | 2X10^-16^ |
| All bee visit male flower  Normal year(2015) | 0.42 | 0.12 | 3.53 | 0.0004 |

**Table S15: Comparison of all bee visitation to all the female flowers between the drought and the normal rainfall year with farm id as a random factor.**

| Poisson family |  |  |  |  |
| --- | --- | --- | --- | --- |
| AIC | BIC | log likelihood | Deviance | Residual_Df |
| 864.3 | 878.2 | -429.1 | 858.3 | 768 |
|  |  |  |  |  |
| Random effects: |  |  |  |  |
|  |  |  |  |  |
| Conditional model: |  |  |  |  |
| Groups | Name | Variance | Standard deviation |  |
| Farm_ID | Intercept | 0.011 | 0.11 |  |
|  |  |  |  |  |
| Number of observation | 771 | Groups: | 24 |  |
|  |  |  |  |  |
|  |  |  |  |  |
|  |  |  |  |  |
| Conditional model: |  |  |  |  |
|  | Estimate | Std.error | z-value | P |
| Intercept | -1.41 | 0.11 | -13.14 | 2X10^-16^ |
| All bee visit female flower  Normal year(2015) | 0.13 | 0.14 | 0.9 | 0.36 |

**Table S16: Comparison of *Trigona* sp. visitation to all flowers between the drought and the normal rainfall year with farm id as a random factor.**

| Poisson family |  |  |  |  |
| --- | --- | --- | --- | --- |
| AIC | BIC | log likelihood | Deviance | Residual_Df |
| 784.8 | 798.7 | -389.4 | 778.8 | 768 |
|  |  |  |  |  |
| Random effects: |  |  |  |  |
|  |  |  |  |  |
| Conditional model: |  |  |  |  |
| Groups | Name | Variance | Standard deviation |  |
| Farm_ID | Intercept | 0.67 | 0.82 |  |
|  |  |  |  |  |
| Number of observation | 771 | Groups: | 24 |  |
|  |  |  |  |  |
|  |  |  |  |  |
|  |  |  |  |  |
| Conditional model: |  |  |  |  |
|  | Estimate | Std.error | z-value | P |
| Intercept | -2.11 | 0.22 | -9.5 | 2X10^-16^ |
| *Trigona* sp. visit all flower Normal year(2015) | 0.75 | 0.15 | 4.79 | 1.6X10^-6^ |

**Table S17: Comparison of *Trigona* sp. visitation to male flowers between the drought and the normal rainfall year with farm id as a random factor.**

| Poisson family |  |  |  |  |
| --- | --- | --- | --- | --- |
| AIC | BIC | log likelihood | Deviance | Residual_Df |
| 562.1 | 576.2 | -278.1 | 556.1 | 793 |
|  |  |  |  |  |
| Random effects: |  |  |  |  |
|  |  |  |  |  |
| Conditional model: |  |  |  |  |
| Groups | Name | Variance | Standard deviation |  |
| Farm_ID | Intercept | 0.57 | 0.75 |  |
|  |  |  |  |  |
| Number of observation | 796 | Groups: | 24 |  |
|  |  |  |  |  |
|  |  |  |  |  |
|  |  |  |  |  |
| Conditional model: |  |  |  |  |
|  | Estimate | Std.error | z-value | P |
| Intercept | -2.66 | 0.24 | -11.04 | 2X10^-16^ |
| *Trigona* sp. visit male flower Normal year(2015) | 0.81 | 0.2 | 4.03 | 5.47X10^-5^ |

**Table S18: Comparison of *Trigona* sp. visitation to female flowers between the drought and the normal rainfall year with farm id as a random factor.**

| Poisson family |  |  |  |  |
| --- | --- | --- | --- | --- |
| AIC | BIC | log likelihood | Deviance | Residual_Df |
| 479.3 | 493.2 | -236.6 | 473.3 | 768 |
|  |  |  |  |  |
| Random effects: |  |  |  |  |
|  |  |  |  |  |
| Conditional model: |  |  |  |  |
| Groups | Name | Variance | Standard deviation |  |
| Farm_ID | Intercept | 0.38 | 0.62 |  |
|  |  |  |  |  |
| Number of observation | 771 | Groups: | 24 |  |
|  |  |  |  |  |
|  |  |  |  |  |
|  |  |  |  |  |
| Conditional model: |  |  |  |  |
|  | Estimate | Std.error | z-value | P |
| Intercept | -2.78 | 0.24 | -11.46 | 2X10^-16^ |
| *Trigona* sp. visit female flower Normal year(2015) | 0.63 | 0.23 | 2.69 | 0.0072 |

**Table S19: Comparison of *Apis dorsata* visitation to all flowers between the drought and the normal rainfall year with farm id as a random factor.**

| Poisson family |  |  |  |  |
| --- | --- | --- | --- | --- |
| AIC | BIC | log likelihood | Deviance | Residual_Df |
| 431.9 | 445.9 | -213 | 425.9 | 768 |
|  |  |  |  |  |
| Random effects: |  |  |  |  |
|  |  |  |  |  |
| Conditional model: |  |  |  |  |
| Groups | Name | Variance | Standard deviation |  |
| Farm_ID | Intercept | 0.093 | 0.31 |  |
|  |  |  |  |  |
| Number of observation | 771 | Groups: | 24 |  |
|  |  |  |  |  |
|  |  |  |  |  |
|  |  |  |  |  |
| Conditional model: |  |  |  |  |
|  | Estimate | Std.error | z-value | P |
| Intercept | -1.99 | 0.16 | -12.64 | 2X10^-16^ |
| *A. dorsata* visit all flower Normal year(2015) | -0.97 | 0.26 | -3.77 | 0.0002 |

**Table S20: Comparison of *Apis dorsata* visitation to male flowers between the drought and the normal rainfall year with farm id as a random factor.**

| Poisson family |  |  |  |  |
| --- | --- | --- | --- | --- |
| AIC | BIC | log likelihood | Deviance | Residual_Df |
| 320.7 | 334.7 | -157.3 | 314.7 | 793 |
|  |  |  |  |  |
| Random effects: |  |  |  |  |
|  |  |  |  |  |
| Conditional model: |  |  |  |  |
| Groups | Name | Variance | Standard deviation |  |
| Farm_ID | Intercept | 3.82X10^-9^ | 6.18X10^-5^ |  |
|  |  |  |  |  |
| Number of observation | 771 | Groups: | 24 |  |
|  |  |  |  |  |
|  |  |  |  |  |
|  |  |  |  |  |
| Conditional model: |  |  |  |  |
|  | Estimate | Std.error | z-value | P |
| Intercept | -2.51 | 0.17 | -14.53 | 2X10^-16^ |
| *A. dorsata* visit male flower Normal year(2015) | -0.77 | 0.31 | -2.44 | 0.015 |

**Table S21: Comparison of *Apis dorsata* visitation to female flowers between the drought and the normal rainfall year with farm id as a random factor.**

| Poisson family |  |  |  |  |
| --- | --- | --- | --- | --- |
| AIC | BIC | log likelihood | Deviance | Residual_Df |
| 218.8 | 232.7 | -106.4 | 212.8 | 768 |
|  |  |  |  |  |
| Random effects: |  |  |  |  |
|  |  |  |  |  |
| Conditional model: |  |  |  |  |
| Groups | Name | Variance | Standard deviation |  |
| Farm_ID | Intercept | 3.12X10^-9^ | 5.59X10^-5^ |  |
|  |  |  |  |  |
| Number of observation | 771 | Groups: | 24 |  |
|  |  |  |  |  |
|  |  |  |  |  |
|  |  |  |  |  |
| Conditional model: |  |  |  |  |
|  | Estimate | Std.error | z-value | P |
| Intercept | -2.83 | 0.21 | -13.53 | 2X10^-16^ |
| *A. dorsata* visit female flower Normal year(2015) | -1.32 | 0.46 | -2.89 | 0.004 |

**Table S22: Comparison of *Apis cerana* visitation to all flowers between the drought and the normal rainfall year with farm id as a random factor.**

| Poisson family |  |  |  |  |
| --- | --- | --- | --- | --- |
| AIC | BIC | log likelihood | Deviance | Residual_Df |
| 570.4 | 584.3 | -282.2 | 564.4 | 768 |
|  |  |  |  |  |
| Random effects: |  |  |  |  |
|  |  |  |  |  |
| Conditional model: |  |  |  |  |
| Groups | Name | Variance | Standard deviation |  |
| Farm_ID | Intercept | 0.29 | 0.54 |  |
|  |  |  |  |  |
| Number of observation | 771 | Groups: | 24 |  |
|  |  |  |  |  |
|  |  |  |  |  |
|  |  |  |  |  |
| Conditional model: |  |  |  |  |
|  | Estimate | Std.error | z-value | P |
| Intercept | -2.57 | 0.21 | -12.01 | 2X10^-16^ |
| *A. cerana* visit all flower Normal year(2015) | 0.88 | 0.21 | 4.27 | 1.97X10^-5^ |

**Table S23: Comparison of *Apis cerana* visitation to male flowers between the drought and the normal rainfall year with farm id as a random factor.**

| Poisson family |  |  |  |  |
| --- | --- | --- | --- | --- |
| AIC | BIC | log likelihood | Deviance | Residual_Df |
| 441.8 | 455.8 | -217.9 | 435.8 | 793 |
|  |  |  |  |  |
| Random effects: |  |  |  |  |
|  |  |  |  |  |
| Conditional model: |  |  |  |  |
| Groups | Name | Variance | Standard deviation |  |
| Farm_ID | Intercept | 0.25 | 0.49 |  |
|  |  |  |  |  |
| Number of observation | 796 | Groups: | 24 |  |
|  |  |  |  |  |
|  |  |  |  |  |
|  |  |  |  |  |
| Conditional model: |  |  |  |  |
|  | Estimate | Std.error | z-value | P |
| Intercept | -3.07 | 0.25 | -12.28 | 2X10^-16^ |
| *A. cerana* visit male flower Normal year(2015) | 1.05 | 0.26 | 4.08 | 4.45X10^-5^ |

**Table S24: Comparison of *Apis cerana* visitation to female flowers between the drought and the normal rainfall year with farm id as a random factor.**

| Poisson family |  |  |  |  |
| --- | --- | --- | --- | --- |
| AIC | BIC | log likelihood | Deviance | Residual_Df |
| 275.8 | 289.8 | -134.9 | 269.8 | 768 |
|  |  |  |  |  |
| Random effects: |  |  |  |  |
|  |  |  |  |  |
| Conditional model: |  |  |  |  |
| Groups | Name | Variance | Standard deviation |  |
| Farm_ID | Intercept | 4.48X10^-9^ | 6.69X10^-5^ |  |
|  |  |  |  |  |
| Number of observation | 771 | Groups: | 24 |  |
|  |  |  |  |  |
|  |  |  |  |  |
|  |  |  |  |  |
| Conditional model: |  |  |  |  |
|  | Estimate | Std.error | z-value | P |
| Intercept | -3.38 | 0.27 | -12.28 | 2X10^-16^ |
| *A. cerana* visit female flower Normal year(2015) | 0.63 | 0.34 | 1.86 | 0.06 |

**Table S25: Comparison of *Apis florea* visitation to all flowers between the drought and the normal rainfall year with farm id as a random factor.**

| Poisson family |  |  |  |  |
| --- | --- | --- | --- | --- |
| AIC | BIC | log likelihood | Deviance | Residual_Df |
| 569 | 583 | -281.5 | 563 | 768 |
|  |  |  |  |  |
| Random effects: |  |  |  |  |
|  |  |  |  |  |
| Conditional model: |  |  |  |  |
| Groups | Name | Variance | Standard deviation |  |
| Farm_ID | Intercept | 0.49 | 0.69 |  |
|  |  |  |  |  |
| Number of observation | 771 | Groups: | 24 |  |
|  |  |  |  |  |
|  |  |  |  |  |
|  |  |  |  |  |
| Conditional model: |  |  |  |  |
|  | Estimate | Std.error | z-value | P |
| Intercept | -2.13 | 0.22 | -9.88 | 2X10^-16^ |
| *A. florea* visit all flower Normal year(2015) | -0.16 | 0.19 | -0.83 | 0.41 |

**Table S26: Comparison of *Apis florea* visitation to male flowers between the drought and the normal rainfall year with farm id as a random factor.**

| Poisson family |  |  |  |  |
| --- | --- | --- | --- | --- |
| AIC | BIC | log likelihood | Deviance | Residual_Df |
| 376 | 390 | -185 | 370 | 793 |
|  |  |  |  |  |
| Random effects: |  |  |  |  |
|  |  |  |  |  |
| Conditional model: |  |  |  |  |
| Groups | Name | Variance | Standard deviation |  |
| Farm_ID | Intercept | 0.19 | 0.44 |  |
|  |  |  |  |  |
| Number of observation | 796 | Groups: | 24 |  |
|  |  |  |  |  |
|  |  |  |  |  |
|  |  |  |  |  |
| Conditional model: |  |  |  |  |
|  | Estimate | Std.error | z-value | P |
| Intercept | -2.65 | 0.22 | -11.95 | 2X10^-16^ |
| *A. florea* visit male flower Normal year(2015) | -0.15 | 0.26 | -0.58 | 0.56 |

**Table S27: Comparison of *Apis florea* visitation to female flowers between the drought and the normal rainfall year with farm id as a random factor.**

| Poisson family |  |  |  |  |
| --- | --- | --- | --- | --- |
| AIC | BIC | log likelihood | Deviance | Residual_Df |
| 360.2 | 374.1 | -177.1 | 354.2 | 768 |
|  |  |  |  |  |
| Random effects: |  |  |  |  |
|  |  |  |  |  |
| Conditional model: |  |  |  |  |
| Groups | Name | Variance | Standard deviation |  |
| Farm_ID | Intercept | 0.36 | 0.59 |  |
|  |  |  |  |  |
| Number of observation | 771 | Groups: | 24 |  |
|  |  |  |  |  |
|  |  |  |  |  |
|  |  |  |  |  |
| Conditional model: |  |  |  |  |
|  | Estimate | Std.error | z-value | P |
| Intercept | -2.75 | 0.26 | -10.71 | 2X10^-16^ |
| *A. florea* visit female flower Normal year(2015) | -0.22 | 0.28 | -0.79 | 0.43 |
|  |  |  |  |  |

**Table S28: GLM with quasibinomial error structure examining the effect of pollinator diversity (Shannon’s diversity index) on FFR in A) drought (2014) and B) normal rainfall**

**year (2015). As expected, owing to presence of all the pollinators in both years, the diversity of bees did not affect the FFR.**

| A) |  |  |  |  |
| --- | --- | --- | --- | --- |
| Deviance residuals |  |  |  |  |
| Min | 1Q | Median | 3Q | Max |
| -2.76 | -0.78 | -0.09 | 0.92 | 2.26 |
|  | Estimate | Std.error | Z value | Pr(>\|z\|) |
| Intercept | -1.52 | 0.48 | -3.14 | 0.005 |
| Bee diversity (2014) | 0.09 | 0.43 | 0.21 | 0.84 |
| Dispersion parameter for quasibinomial family taken to be 1.759172 | | | | |
| Null deviance: | 40.59 on | 22 df |  |  |
| Residual deviance: | 40.52 on | 21 df |  |  |
| B) |  |  |  |  |
| Deviance residuals |  |  |  |  |
| Min | 1Q | Median | 3Q | Max |
| -1.60 | -0.68 | -0.07 | 0.34 | 2.18 |
|  | Estimate | Std.error | Z value | Pr(>\|z\|) |
| Intercept | -0.94 | 0.18 | -5 | 5.21 X 10^-5^ |
| Bee diversity (2015) | 0.34 | 0.21 | -1.66 | 0.11 |
| Dispersion parameter for quasibinomial family taken to be 1.005645 | | | | |
| Null deviance: | 24.96 on | 23 df |  |  |
| Residual deviance: | 22.19 on | 22 df |  |  |

**Table S29: GLM with binomial error structure examining the impact of bee visitation on FFR during A) normal, and B) drought year. The model was built using all bee visitation and their interactions as predictor variables, and FFR as the response variable (in binomial format). The results shown here are after a series of model simplifications by removing non-significant terms.**

| A) |  |  |  |  |
| --- | --- | --- | --- | --- |
| Deviance residuals |  |  |  |  |
| Min | 1Q | Median | 3Q | Max |
| -1.69 | -0.66 | -0.06 | 0.40 | 1.83 |
|  | Estimate | Std.error | Z value | Pr(>\|z\|) |
| Intercept | -1.41 | 0.11 | -13.45 | <2 X 10^-16^ |
| *Trigona* sp. Visitation | 0.46 | 0.22 | 2.12 | 0.034 |
|  |  |  |  |  |
| Null deviance: | 24.96 on | 23 df |  |  |
| Residual deviance: | 20.53 on | 22 df |  |  |
| B) |  |  |  |  |
| Deviance residuals |  |  |  |  |
| Min | 1Q | Median | 3Q | Max |
| -1.95 | -0.77 | -0.008 | 0.81 | 1.75 |
|  | Estimate | Std.error | Z value | Pr(>\|z\|) |
| Intercept | -2.06 | 0.18 | -11.20 | <2 X 10^-16^ |
| *Apis dorsata* visitation | 3.31 | 0.85 | 3.92 | 9.06 X 10^-05^ |
| *Trigona* sp. visitation | 0.85 | 0.43 | 2.00 | 0.046 |
| Null deviance: | 40.60 on | 22 df |  |  |
| Residual deviance: | 24.74 on | 20 df |  |  |

|  |  |  |  |  |  |  |  |
| --- | --- | --- | --- | --- | --- | --- | --- |

**Table S30: GLM with quasibinomial error structure (due to overdispersion) examining the impact of bee visitation on FFR during drought year (2014) after removing the farmland data point with very high *Trigona* sp. visitation (0.85). The model was built using all bee visitation and their interactions as predictor variables, and FFR as the response variable (in binomial format). The results shown here are after a series of model simplifications by removing non-significant terms.**

| Deviance residuals |  |  |  |  |
| --- | --- | --- | --- | --- |
| Min | 1Q | Median | 3Q | Max |
| -2.53 | -0.41 | 0.05 | 0.32 | 1.39 |
|  | Estimate | Std.error | Z value | Pr(>\|z\|) |
| Intercept | -2.88 | 0.37 | -7.68 | 9.4 X 10^-7^ |
| *A. dorsata* Visitation | 6.01 | 1.50 | 4.01 | 0.001 |
| *A. florea* Visitation | 6.17 | 2.65 | 2.32 | 0.03 |
| *Trigona* sp. Visitation | 4.97 | 1.86 | 2.67 | 0.01 |
| Interaction (*A. dorsata* and *A. florea*) | -22.44 | 9.13 | -2.45 | 0.03 |
| Interaction (*Trigona* sp. and *A. florea*) | -26.09 | 11.73 | -2.22 | 0.04 |
| (Dispersion parameter for quasibinomial family taken to be 0.8899445) | | | | |
| Null deviance: | 40.51 on | 21 df |  |  |
| Residual deviance: | 15.55 on | 16 df |  |  |

**Table S31: Wilcoxon signed rank test (one tailed) examining if *Apis dorsata* visitation was significantly higher in each farmland, and *Trigona* sp. visitation was significantly lower in each farmland during drought year in comparison to the normal rainfall year. The farmlands where *Trigona* sp. decline was concomitant with *Apis dorsata* increase are highlighted in bold. A total of 19 farmlands showed increase in *Apis dorsata* visitation, and 18 farmlands showed decline in *Trigona* sp. visitation during drought.**

|  | ***Apis dorsata*** | | ***Trigona* sp.** | |
| --- | --- | --- | --- | --- |
| **Farmno** | **Test statistic (W)** | **p value** | **Test statistic (W)** | **p value** |
| Field1 | 50 | 0.99 | 7 | 6.97x10^-6^ |
| Field11 | 283.5 | **0.005** | 121 | **0.02** |
| Field14 | 276 | **2.15x10^-6^** | 10 | **1.06x10^-6^** |
| Field17 | 126 | **2.85x10^-5^** | 3 | **9.51x10^-6^** |
| Field19 | 128 | **4.7x10^-5^** | 18 | **0.001** |
| Field2 | 173.5 | 0.59 | 9 | 7.13x10^-8^ |
| Field22 | 226 | 4.34x10^-5^ | 252 | 1 |
| Field25 | 236 | **1.77x10^-5^** | 32 | **6.47x10^-5^** |
| Field28 | 234.5 | 3.22x10-5 | 248 | 1 |
| Field30 | 365 | 8.71x10^-7^ | 170 | 0.27 |
| Field4 | 117 | 0.97 | 206 | 0.98 |
| Field6 | 245.5 | **5.07x10^-6^** | 39 | **0.0004** |
| Field7 | 256 | **0.03** | 90 | **0.002** |
| FieldA | 357 | **2.27x10^-7^** | 53.5 | **6.89x10^-5^** |
| FieldF | 230 | **6.5x10^-5^** | 60 | **0.005** |
| FieldF33 | 256 | **1.4x10^-7^** | 6 | **4.99x10^-8^** |
| FieldK | 126 | 2.02x10^-5^ | 128 | 1 |
| FieldL | 166 | **0.0005** | 6 | **9.86x10^-7^** |
| FieldM | 119.5 | 0.63 | 0 | 1.66x10^-9^ |
| FieldN2 | 254.5 | 0.02 | 345 | 1 |
| FieldO | 33 | 0.97 | 32 | 0.01 |
| FieldP11 | 92 | **0.04** | 0 | **3.72x10^-5^** |
| FieldSN14 | 225 | **0.0001** | 11 | **4.5x10^-6^** |
| FieldZ | 254 | **6.88x10^-7^** | 5 | **3.16x10^-8^** |
